# Maternal Mental Health in Late Pregnancy and Longitudinal Changes in Postpartum Serum Vitamin B-12, Homocysteine, and Milk B-12 Concentration Among Brazilian Women

**DOI:** 10.3389/fnut.2022.923569

**Published:** 2022-07-11

**Authors:** Mônica Araujo Batalha, Paula Normando dos Reis Costa, Ana Lorena Lima Ferreira, Nathalia C. Freitas-Costa, Amanda C. Cunha Figueiredo, Setareh Shahab-Ferdows, Daniela Hampel, Lindsay H. Allen, Rafael Pérez-Escamilla, Gilberto Kac

**Affiliations:** ^1^Nutritional Epidemiology Observatory, Josué de Castro Nutrition Institute, Rio de Janeiro Federal University, Rio de Janeiro, Brazil; ^2^United States Department of Agriculture/Agricultural Research Service, Western Human Nutrition Research Center, Davis, CA, United States; ^3^Department of Nutrition, University of California, Davis, Davis, CA, United States; ^4^Department of Social and Behavioral Sciences, Yale School of Public Health, New Haven, CT, United States

**Keywords:** pregnancy, lactation, human milk, vitamin B12, homocysteine, anxiety, depression

## Abstract

**Background:**

Little is known regarding the association between mental health distress during pregnancy and postpartum maternal serum biomarkers of vitamin B-12 status and milk B-12 concentration.

**Objective:**

To evaluate the association between depressive and anxiety symptoms in the third trimester of pregnancy and changes in postpartum serum B-12, homocysteine, and B-12 milk concentration.

**Methods:**

A total of 101 women (18–40 years) were studied in a prospective cohort with data at the third trimester of pregnancy (baseline) and three postpartum time-points (TPs): 2–8 days (TP1), 28–50 days (TP2), and 88-119 days (TP3) postpartum. B-12 concentrations in milk were measured by competitive chemiluminescent enzyme immunoassay at TP1, TP2, and TP3. Serum B-12 and homocysteine concentrations were evaluated at baseline, TP1, TP2, and TP3 by chemiluminescent immunoassays. Depressive and anxiety symptoms were measured with the Edinburgh Postnatal Depression Scale and the State-Trait Anxiety Inventory at baseline. Spearman's correlation test and multiple linear mixed-effect models were performed.

**Results:**

The prevalence of depressive and anxiety state symptoms was 35.6 and 39.6% at baseline. High prevalence of low milk B-12 concentration (<310 pmol/L) were observed at TP1 (53.2%), TP2 (71.4%), and TP3 (71.1%). Women with anxiety symptoms at baseline presented higher median concentrations of serum homocysteine at TP1 and lower concentrations of serum and milk B-12 at TP2 compared with women without anxiety symptoms [8 (7; 9) *vs*. 6 (5; 8) and 266 (188; 369) *vs*. 332 (272; 413)]. Milk B-12 concentrations were positively and significantly correlated with maternal serum B-12 concentrations at different TP. Women with anxiety symptoms at baseline exhibited a decrease in daily postpartum homocysteine concentrations compared to women without anxiety symptoms (β = −0.002, SE = 0.001, *p* = 0.024).

**Conclusion:**

Anxiety symptoms at the end of pregnancy were associated with longitudinal changes in maternal serum homocysteine concentrations during the first 3 months postpartum.

## Introduction

Vitamin B12 (B-12) is an essential nutrient for maternal and child health. It participates as a cofactor in various metabolic processes, including the one-carbon cycle, which is essential for synthesizing DNA and amino acids (1, 2). Women of childbearing age, infants, and young children are included in the group at higher risk for B-12 insufficiency (3, 4). B-12 deficiency during pregnancy has been linked to adverse gestational outcomes (e.g., preterm birth and low birth weight) and could impact the infants' fetal storage of this vitamin (3, 5–7).

There is substantial interest in the relationship between maternal nutrition and maternal mental health in the perinatal period (8). Some studies have linked inadequate intake and low-status biomarkers of folate and B-12, vitamins involved in the one-carbon cycle, in subjects with depressive and anxiety symptoms (9–12). However, these studies were cross-sectional and mixed results were observed (4, 10). B-12 has hematological and neurological functions and is required for S-adenosylmethionine (SAM) synthesis (4), while SAM has anti-depressive properties, as it plays a role in synthesizing neurotransmitters (13). Some studies have demonstrated that B-12 deficiency status could impair SAM synthesis and raise homocysteine levels, thus being related to anxiety and depressive symptoms (14, 15). Homocysteine is a sulfur-containing amino acid formed during the methionine metabolism that also plays a role in neurotransmitter synthesis (16, 17). Hyperhomocysteinemia has been linked to both depression and cardiovascular disease, although results remain inconsistent (17).

Mental health disorders have been linked to micronutrient deficiencies, including B-12 deficiency, as they could contribute to unhealthy lifestyles (18–20), however, this relationship has been less investigated. Thus, this area is of major clinical and public health concern, given the high prevalence of mental health disorders during pregnancy in low- and middle-income countries, in which the prevalence of prenatal depression is around 25.8% (21). In addition, anxiety symptoms seem to increase through pregnancy trimesters, and a prevalence of approximately 24.6% had been self-reported in the third trimester (22).

Perinatal depressive and anxiety are related to several undesirable maternal and child health outcomes, such as poor dietary intake, increased incidence of preterm birth, low Apgar score, and could have a negative impact on breastfeeding practices (19, 23, 24). However, few studies have assessed the association of maternal mental health with human milk composition. Previous studies have focused on mental health disorders during the postpartum period concerning hormones and immunological factors in milk as a physiological response to stress (23, 25). Nevertheless, the relationship between maternal mental health during pregnancy and postpartum B-12 and homocysteine concentration is not well-established, and little is known about their association with milk B-12 concentration. This study tests the primary hypotheses that depressive or anxiety symptoms during the third trimester of pregnancy are associated with changes in maternal serum B-12 and homocysteine concentrations and milk B-12 concentrations during the postpartum period. Additionally, we tested if (1) medians of serum B-12 and homocysteine concentrations and milk B-12 concentrations were different according to maternal mental health status; (2) milk B-12 concentrations are positively correlated with maternal serum B-12 and negatively correlated with maternal homocysteine concentrations.

## Materials and Methods

Women and their infants participating in this prospective cohort study received perinatal care at a public health center located in a lower-income community in Rio de Janeiro, Brazil. The study data were collected between January 2017 and January 2020. Eligibility criteria comprised pregnant mothers between 28 and 35 gestational weeks; between 18 and 40 y; with no known infectious or chronic non-communicable diseases (except obesity); singleton pregnancy; and residence in the catchment area of the public health center.

A non-probabilistic sample of 147 pregnant women was enrolled during the study baseline when women were between 28 and 35 weeks of gestation. Subsequently, eight women and their children were excluded due to stillbirth, pre-term birth, or developing a chronic or infectious disease before delivery. As a result, 139 women were followed at five follow-up visits that happened at the following time points (TPs) postpartum: 2–8 days (TP1), 28–50 days (TP2), 88–119 days (TP3), 6 months (TP4), and 12 mo (TP5). The TP postpartum visits were defined considering the routine of maternal and infant health care preconized by the Brazilian Health Ministry. Some refused to answer mental health questionnaires (*n* = 4) or collect blood/milk biological samples (*n* = 34). Therefore, we analyzed data from the subsample of 101 women for which we had mental health data and postpartum biological (blood or milk) samples for at least TP1, TP2, and TP3 (Supplementary Figure 1).

### Maternal Mental Health

Depressive symptoms were measured at the baseline with the Edinburgh Postnatal Depression Scale (EPDS), the most common screening tool used in the perinatal period (26, 27). EPDS is a 10-item screening questionnaire that assesses depressive symptoms over the preceding 7 days (28, 29). Each item in this scale has four possible answers with qualitative frequency options (e.g., yes, most of the time, or not at all). The options range from 0 to 3, with a maximum score of 30. We classified the participants as with (EPDS ≥11) or without (EPDS <11) depressive symptoms at baseline.

Anxiety symptoms were measured at the baseline with the Spielberger State-Trait Anxiety Inventory (STAI) which evaluates anxiety traits and a state, two different anxiety types (30). The anxiety trait refers to stable and relatively permanent personality characteristics. The anxiety state refers to a transitory emotional state concerning a specific period. The STAI consists of 40 items separated into two distinct subscales of 20 items each, with Likert-type response options ranging from 1 to 4. The total STAI score for each subscale (state-anxiety and trait-anxiety) ranges from 20 to 80 (30). In the present study, we used an anxiety state to evaluate the anxiety related to the specific moment of pregnancy (in the third trimester). We classified the participants as with (STAI ≥40) or without (STAI <40) anxiety symptoms.

The EPDS and STAI instruments were validated for use in the pregnancy and postnatal periods (29, 31). The cross-cultural adaptation of STAI comprised four steps previously described (32), and then, the instrument was translated and adapted for use in Brazil (33). The Portuguese version of EPDS was validated in a study conducted by Santos et al. (34).

### Maternal Serum B-12 and Homocysteine

After an 8-12 h overnight fast, maternal blood samples were collected at baseline, TP1, TP2, and TP3. The samples were collected in tubes with separator gel and centrifuged within 1 h of blood collection. Serum was separated into aliquots of 1 ml and stored at −80°C until analysis. Serum B-12 concentrations were analyzed by chemiluminescent immunoassays in an automated system (UniCel DxI 800, Beckman Coulter, Brea, USA), and homocysteine concentrations were analyzed by chemiluminescent immunoassays in an Abbott Alinity i (Abbott Diagnostics, Chicago, USA). B-12 deficient status was defined as serum level <148 pmol/L (35) and serum homocysteine >15 μmol/L was defined as hyperhomocysteinemia (36). These cut points were only used to characterize the participants' status in the postpartum period, as they overestimate the actual prevalence of B-12 deficiency in pregnancy (4). In our models, serum B-12 and homocysteine were used as continuous variables.

### Human Milk Collection and Milk B-12 Concentration Analysis

Following procedures established by the Brazilian Network of Human Milk Banks protocol (37), we instructed women breastfeeding on-demand to collect milk samples at TP1, TP2, and TP3, as previously described (38). The participants collected milk, preferably in the morning after breakfast (until ~10:00 am), considering that the concentrations of B vitamins in milk exhibit little or no diurnal variation (39). The milk samples were collected from the same breast through hand expression because a pump could be a potential risk of contamination. Before starting the collection, the participants were asked to wash their hands and put on masks to protect their face and head coverings to avoid possible sample contamination during the process. The trained interviewer also used the same hygiene procedures because, if necessary, they helped the participant with the milk sample extraction using nitrile gloves.

In total, 5 ml of milk was collected from each participant at TP1 and 17 ml at TP2 and TP3. The samples were expressed directly into 50 ml sterile, ribonuclease (RNase)-free, deoxyribonuclease (DNase)-free, and non-pyrogenic tubes. Although the breast was not fully emptied, previous studies have shown that the concentrations of water-soluble vitamins in milk remain relatively stable during the feeding episode (hind or foremilk) (39). The samples were homogenized and aliquoted immediately after collection. One aliquot of 1 ml at each visit was used for B-12 analyses. The samples were frozen at −20°C and then transported in a temperature-controlled box (−1 to −5°C) to Rio de Janeiro Federal University and stored at −80°C. For analysis, the frozen samples were shipped on dry ice to the USDA/ARS-Western Human Nutrition Research Center, Davis, CA, USA. Milk B-12 was analyzed using the Siemens IMMULITE 1000 competitive chemiluminescent enzyme immunoassay (coefficient of variation: 8.5%) as previously described (40). To evaluate the prevalence of low B-12 milk concentration, we consider a cutoff of <310 pmol/L proposed by Williams et al. (41). In our models, milk B-12 was used as a continuous variable.

### Covariates

A structured questionnaire was administered at baseline to collect socio-economic and demographic data, such as maternal age (years), education (schooling years), marital status (with and without a partner), and parity (primiparous/multiparous). Breastfeeding status was defined as exclusive and predominant (42). Exclusive breastfeeding means no other food or drink, not even water, except human milk for the first 6 months of life, except for rehydration solution, drops, and syrups (vitamins, minerals, and medicines). Predominant breastfeeding means that the infant's primary source of nourishment is human milk; however, the infant may also have received liquids (including water or water-based drinks and fruit juice), ritual fluids, and medicines (42).

Serum folate concentrations (ng/ml) were analyzed by chemiluminescent immunoassay in an automated system UniCel DxI 800 (Beckman Coulter, Brea, USA).

The interviewers were trained to measure height in duplicate using a stadiometer (Altura Exata, Belo Horizonte, Brazil) at TP2. When the measurements of height differed by >0.5 cm, a third measurement was performed, and the mean of the two more similar measurements was used. The self-reported pre-pregnancy weight was used to calculate the pre-pregnancy BMI (kg/m^2^); women were classified according to the WHO cutoffs (43) as underweight (<18.5 kg/m^2^), normal (≥18.5 and <25.0 kg/m^2^), overweight (≥25.0 and <30.0 kg/m^2^), or obese (≥30 kg/m^2^).

### Statistical Analyses

The distribution of continuous variables was assessed using histograms, kurtosis, and skewness measurements. We used absolute (*n*) and relative frequencies (%) for categorical variables and median and interquartile range (IQR) for continuous variables to describe maternal characteristics, blood biomarkers, and milk B-12 concentrations. We compared the characteristics of women with and without biological samples using the Mann–Whitney, Chi-square, and Fisher's exact tests.

The Mann–Whitney test was performed to compare maternal serum B-12, homocysteine, and milk B-12 median concentrations, at each visit, between women with or without anxiety and depressive symptoms. The Spearman's rank correlation test was performed to investigate correlations between maternal B-12 and serum homocysteine concentrations during pregnancy and lactation and milk B-12 at TP1, TP2, and TP3. The correlations were interpreted as weak (0.00 to 0.39), moderate (0.40 to 0.69), strong (0.70 to 0.89), and very strong (0.90 to 1.00) (44).

Unadjusted and adjusted longitudinal linear mixed-effects models were performed using baseline anxiety or depressive symptom scores as interaction terms. These models were performed to evaluate whether maternal mental health (exposures) were associated with daily postpartum changes in serum B-12 and homocysteine and milk B-12 concentrations (outcomes). Considering that our outcomes were skewed, the regression models used log-transformed variables. We performed diagnostic plots to check for violations of the model's assumptions; residual plots were used to assess the linearity and homoscedasticity. Quantile-quantile plots were used to check the normality of the residuals and random effects.

All the models were adjusted for specific confounders selected from previously published studies. The models in which postpartum serum B-12 and homocysteine were the outcomes were adjusted for maternal age, education, pre-pregnancy BMI, B-vitamin supplement intake during pregnancy (including folate), and serum folate concentration in the third trimester of pregnancy. The model in which postpartum milk B-12 was the outcome was adjusted for pre-pregnancy BMI, B-vitamin supplement intake during pregnancy (including folate), and serum B-12 concentration in the third trimester of pregnancy. The associations were considered statistically significant when *p*-values were <0.05. Statistical analyses were performed using Stata version 15 (StataCorp) and R version 4.0.3.

## Results

The median maternal age was 26 (IQR: 22–31) years, and 44.7% of the participants were classified as pre-pregnancy overweight or obese. The prevalence of depressive and anxiety state symptoms was 35.6 and 39.6% at baseline, respectively. The prevalence of B-12 deficient status (<148 pmol/L) at TP1 was 27.3% and 3.9% at TP2, and no case of hyperhomocysteinemia (>15 μmol/L) was observed during the postpartum period. The prevalence of low milk B-12 concentrations (<310 pmol/L) was 53.2% at TP1, 71.4% at TP2, and 71.1% at TP3, respectively (Table 1). The participants with and without biological (milk and blood) samples were not statistically different when comparing socio-economic, demographic, and biomedical characteristics at baseline (Supplementary Table 1).

**Table 1:** Characteristics, biomarkers, and human milk concentrations of participants followed in the cohort in Rio de Janeiro, Brazil.

**Characteristic**	**Values^a^**
Maternal age (*n* = 101)	26 (22; 31)
Education (years) (*n* = 100)	12 (9; 12)
**Parity (*****n*** **= 94)**	
Primiparous	51 (54.3)
Multiparous	43 (45.7)
**Marital status (*****n*** **= 101)**	
With a partner	84 (83.2)
Without a partner	17 (16.8)
**Pre-pregnancy BMI (kg/m**^2^**) (*****n*** **= 94)**	
Underweight (<18.5)	3 (3.2)
Normal weight (18.5–24.9)	49 (52.1)
Overweight (25.0–29.9)	30 (31.9)
Obesity (≥30.0)	12 (12.8)
**B-vitamin supplement intake during pregnancy (*****n*** **= 101)**^b^	
Yes	89 (88.1)
No	12 (11.9)
**Multivitamin supplement intake during postpartum (*****n*** **= 101)**	
Yes	3 (3.0)
No	98 (97.0)
**Maternal mental health at 3rd trimester of pregnancy**	
Edinburgh Postnatal Depression Scale scores (*n* = 101)	9 (6;12)
**Edinburgh Postnatal Depression Scale categories (*****n*** **= 101)**	
≥11	36 (35.6)
<11	65 (64.4)
State-Trait Anxiety Inventory scores (*n* = 101)	37 (33; 42)
**State-Trait Anxiety Inventory categories (*****n*** **= 101)**	
≥40	40 (39.6)
<40	61 (60.4)
**Serum biomarkers at 3rd trimester of pregnancy**	
B-12 (pmol/L) (=92)	172 (142; 220)
Homocysteine (μmol/L) (*n* = 91)	5 (4; 5)
Folate (ng/Ml) (*n* = 92)	10 (8; 14)
**Serum biomarkers at 2–8 d postpartum**	
B-12 (pmol/L) (*n* = 33)	211 (147; 287)
B-12 deficient status (<148 pmol/L) (*n* = 33)	9 (27.3)
Homocysteine (μmol/L) (*n* = 33)	7 (6; 9)
Hyperhomocysteinemia (>15 μmol/L) (*n* = 33)	0 (0)
**Serum biomarkers at 28–50 d postpartum**	
B-12 (pmol/L) (*n* = 78)	310 (249; 391)
B-12 deficient status (<148 pmol/L) (*n* = 78)	3 (3.9)
Homocysteine (μmol/L) (*n* = 78)	7 (6; 8)
Hyperhomocysteinemia (>15 μmol/L) (*n* = 78)	0 (0)
**Serum biomarkers at 88**–**119 d postpartum**	
B-12 (pmol/L) (*n* = 51)	283 (217; 374)
B-12 deficient status (<148 pmol/L) (*n* = 51)	0 (0)
Homocysteine (μmol/L) (*n* = 51)	6 (6; 8)
Hyperhomocysteinemia (>15 μmol/L) (*n* = 51)	0 (0)
**B-12 human milk concentrations at various time points**	
At 2–8 d postpartum (*n* = 47)	270 (199; 407)
Low concentration (<310 pmol/L) at 2–8 d postpartum (*n* = 47)	25 (53.2)
At 28-50 d postpartum (*n* = 70)	239 (184; 345)
Low concentration (<310 pmol/L) at 28–50 d postpartum (*n* = 70)	50 (71.4)
At 88-119 d postpartum (*n* = 38)	245 (155; 317)
Low concentration (<310 pmol/L) at 88-119 d postpartum (*n* = 38)	27 (71.1)
**Breastfeeding status** ^c^	
Exclusive at 2–8 d postpartum (*n* = 47)	42 (89.4)
Exclusive or predominant at 28–50 d postpartum (*n* = 70)	53 (75.7)
Exclusive or predominant at 88–119 d postpartum (*n* = 38)	26 (68.4)

a *Values are medians (interquartile range) or the number of participants (%)*.

b *Including folic acid supplement intake*.

c *Exclusive breastfeeding is defined as no other food or drink except human milk. Predominant breastfeeding is defined as human milk as an infant's primary source of nourishment; however, the infant may also have received liquids (including water or water-based drinks and fruit juice), ritual fluids, and medicines (42)*.

**Figure 1:**
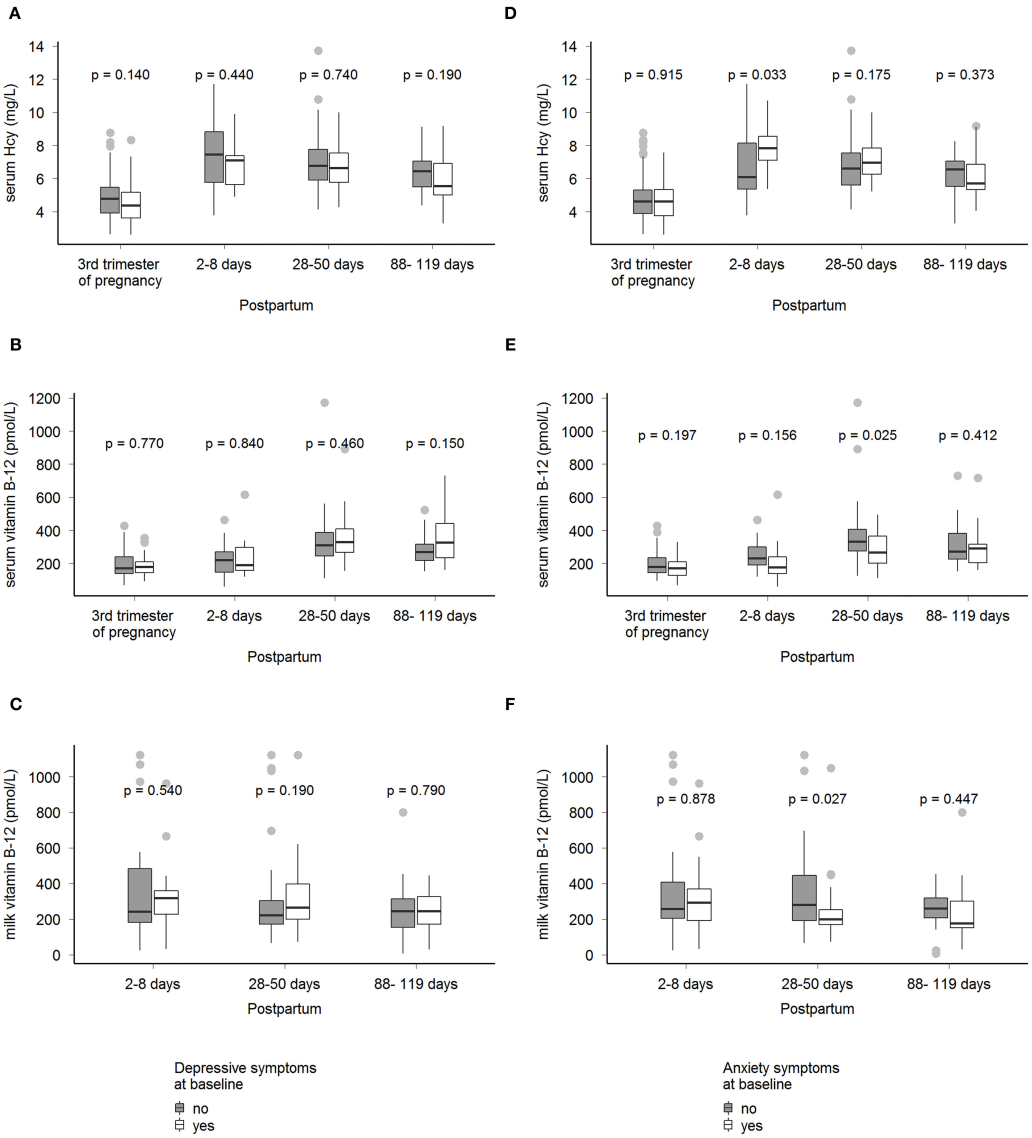
Median concentrations of serum B-12, serum homocysteine, and milk B-12 by maternal mental health status in Rio de Janeiro, Brazil. Mann–Whitney's test was performed to compare medians (IQR) of biological samples (serum vitamin B-12, serum homocysteine, and milk vitamin B-12) at the 3rd trimester of pregnancy, 2-8 d, 28-50 d, and 88-119 d postpartum between women with and without depressive or anxiety symptoms at 3rd trimester of pregnancy (baseline), *p* < 0.05. Depressive symptoms were assessed by the Edinburgh Postnatal Depression Scale (EPDS), yes (EPDS ≥11, *n* = 36) and no (EPDS <11, *n* = 65) **(A–C)**. Anxiety state was assessed by the State-Trait Anxiety Inventory (STAI), yes (STAI ≥40, *n* = 40), and no (STAI <40, *n* = 61) **(D–F)**. Hcy, homocysteine.

Women with anxiety symptoms in the third trimester of pregnancy presented higher median serum homocysteine concentrations at 2–8 days postpartum and lower median concentration of maternal serum and milk B-12 at 28-45 days postpartum compared with women without anxiety symptoms [8 (7; 9) *vs*. 6 (5; 8), *p* = 0.03 and 266 (188; 369) *vs*. 332 (272; 413), *p* = 0.02]. No difference was observed in serum B-12, homocysteine, and B-12 milk concentration between women with and without depressive symptoms at baseline (Figure 1).

### Correlation Between Serum B-12 and Homocysteine Concentrations and Milk B-12 Concentration

The correlations varied between low and moderate. Milk B-12 concentrations at TP1 were positively and significantly correlated with serum B-12 concentrations at baseline (ρ = 0.39, *p* < 0.01) and at TP1 (ρ = 0.64, *p* < 0.01). Milk B-12 concentrations at TP2 were positively and significantly correlated with serum B-12 concentrations at TP1 (ρ = 0.44, *p* = 0.03). Milk B-12 concentrations at TP2 were positively and significantly correlated with serum B-12 levels at TP1 (ρ = 0.44, *p* =0.03) and were negatively and significantly correlated with homocysteine at TP2 (ρ = −0.34, *p* =0.01). Milk B-12 concentrations at TP3 were positively and significantly correlated with serum B-12 levels at TP1 (ρ = 0.64, *p* = 0.02), at TP2 (ρ= 0.48, *p* =0.01), and at TP3 (ρ = 0.39, *p* = 0.02) (data not shown).

### Maternal Mental Health and Changes in the Serum B-12 and Homocysteine, and Milk B-12 Concentrations

**Figure 2:**
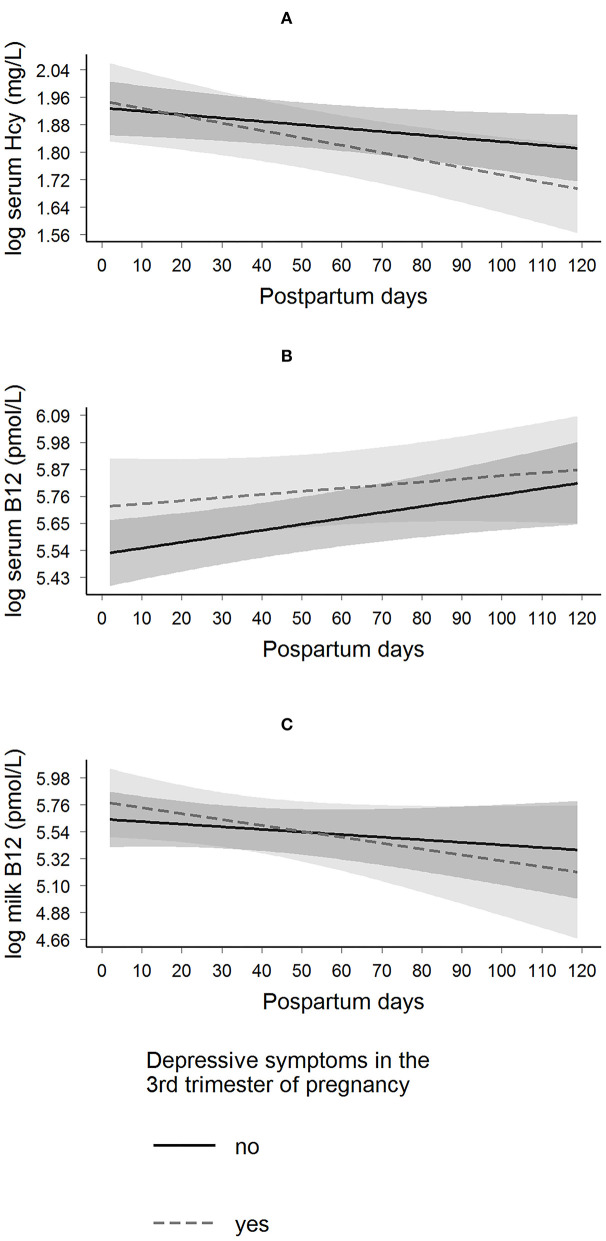
Longitudinal predictions of maternal serum homocysteine, serum B-12, and milk B-12 concentrations according to the presence of depressive symptoms in the 3rd trimester of pregnancy in Rio de Janeiro, Brazil. β Coefficient interactions (β_interaction_) and standard error (SE) were estimated. These parameters were used to evaluate the changes of having depressive symptoms on the trajectory of each biological sample. Depressive symptoms were assessed by the Edinburgh Postnatal Depression Scale (EPDS), yes (EPDS ≥11, *n* = 36) and no (EPDS <11, *n* = 65). **(A)** Log serum homocysteine: β_interaction_ = −0.001 (0.001); *p* = 0.194, and the model was adjusted for maternal age, education, pre-pregnancy BMI, B-vitamin (including folate) supplement intake during pregnancy, and serum folate concentration at the 3rd trimester of pregnancy. **(B)** Log serum B-12: β_interaction_ = −0.001 (0.002); *p* = 0.445, and the model was adjusted for maternal age, education, pre-pregnancy BMI, B-vitamin (including folate) supplement intake during pregnancy, and serum folate concentration at the 3rd trimester of pregnancy **(C)** Log milk-B-12: β_interaction_ = −0.003 (0.004); *p* = 0.455, and the model was adjusted for pre-pregnancy BMI, B-vitamin (including folate) supplement intake during pregnancy, and serum B-12 concentration at the 3rd trimester of pregnancy. Hcy, homocysteine.

**Figure 3:**
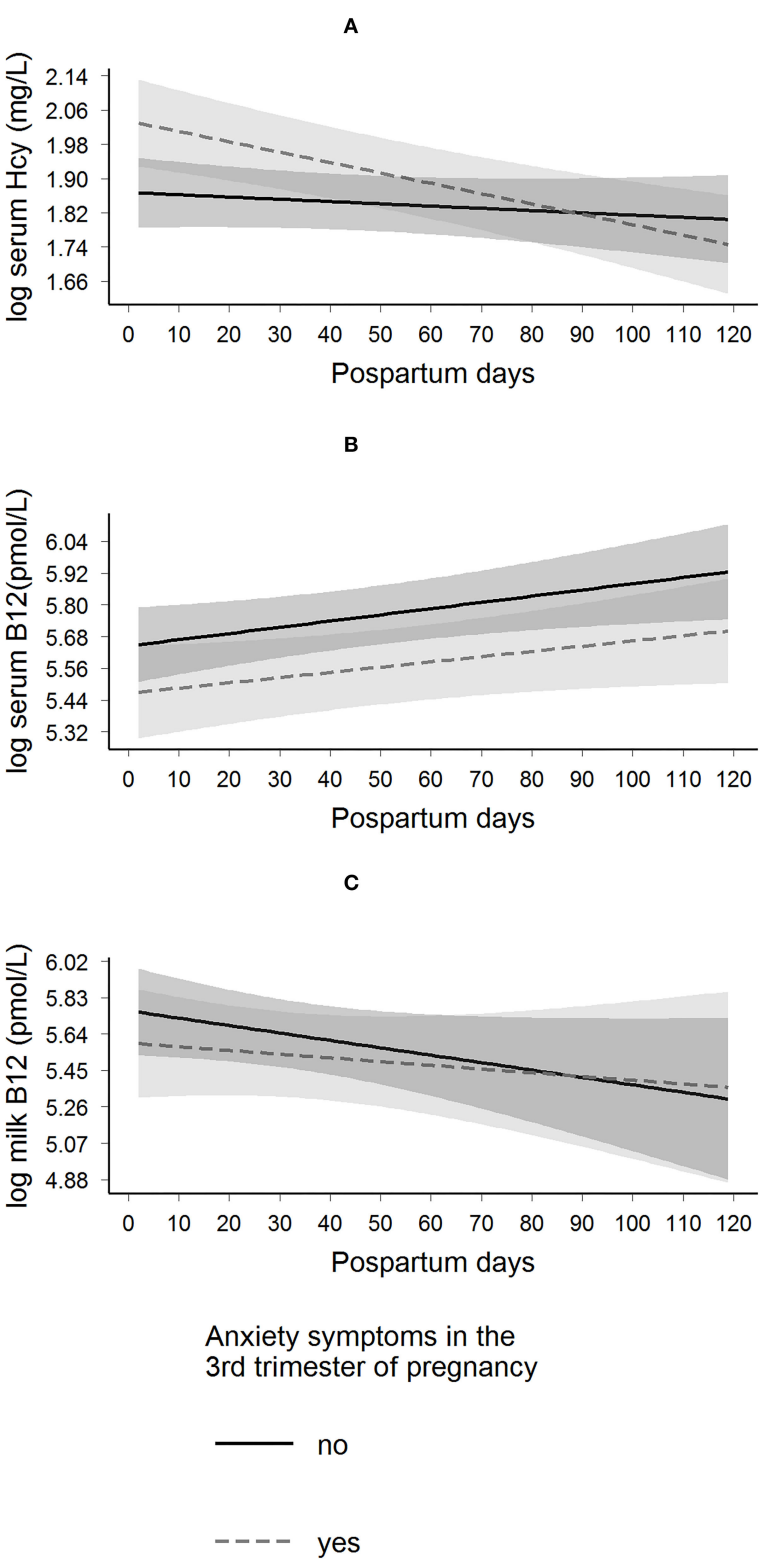
Longitudinal predictions of maternal serum homocysteine, serum B-12, and milk B-12 concentrations according to the presence of anxiety symptoms in the 3rd trimester of pregnancy in Rio de Janeiro, Brazil. β Coefficient interactions (β_interaction_) and standard error (SE) were estimated. These parameters were used to evaluate the changes of having anxiety symptoms on the trajectory of each biological sample. Anxiety state was assessed by the State-Trait Anxiety Inventory (STAI), yes (STAI ≥40, *n* = 40) and no (STAI <40, *n* = 61). **(A)** Log serum homocysteine: β_interaction_ = −0.002 (0.001); *p* = 0.024, and the model was adjusted for maternal age, education, pre-pregnancy BMI, B-vitamin (including folate) supplement intake during pregnancy, and serum folate concentration at the 3rd trimester of pregnancy. **(B)** Log serum B-12: β_interaction_ = −0.000 (0.001); *p* = 0.797, and the model was adjusted for maternal age, education, pre-pregnancy BMI, B-vitamin (including folate) supplement intake during pregnancy, and serum folate concentration at the 3rd trimester of pregnancy **(C)** Log milk-B-12: β_interaction_ = 0.002 (0.004); *p* = 0.585, and the model was adjusted for pre-pregnancy BMI, B-vitamin (including folate) supplement intake during pregnancy, and serum B-12 concentration at the 3rd trimester of pregnancy. Hcy, homocysteine.

The presence of depressive symptoms in the third trimester of pregnancy was not associated with daily postpartum changes in maternal serum B-12, homocysteine, and milk B-12 concentrations (Figure 2 and Table 2). However, women with anxiety symptoms in the third trimester exhibited a decrease in daily postpartum homocysteine concentrations compared to women without anxiety symptoms (β = −0.002 (SE = 0.001), *p* = 0.035), even after adjusting for maternal age, education, pre-pregnancy BMI, B-vitamin supplement intake during pregnancy, and serum folate concentration at the third trimester of pregnancy (β = −0.002 (SE = 0.001), *p* = 0.024) (Figure 3 and Table 3).

**Table 2:** Models of longitudinal prediction of serum homocysteine and vitamin B-12 and milk B-12 trajectories based on the presence of depressive symptoms at the 3^rd^ trimester of pregnancy, Rio de Janeiro, Brazil.

**Unadjusted model**	**Log serum homocysteine (mg/ L) (n = 90)**		**Log serum B-12 (pmol/L) (n = 90)**		**Log milk B-12 (pmol/L) (n = 94)**	
	***β*** **(SE)**^a^	***P***	***β*** **(SE)**^a^	***P***	***β*** **(SE)**^a^	***P***
**Main effects**						
With vs. without depressive symptoms at 3^rd^ trimester^b^	0.013 (0.066)	0.839	0.136 (0.112)	0.228	0.150 (0.182)	0.412
Postpartum days	−0.001 (0.000)	0.046	0.000 (0.001)	0.008	−0.003 (0.002)	0.133
**Interaction term** Postpartum days # with vs. without depressive symptoms at 3^rd^ trimester	−0.001 (0.001)	0.232	−0.000 (0.000)	0.775	−0.002 (0.004)	0.594
**Adjusted model**	***β*** **(SE)**^a^	***P*** ^c^	***β*** **(SE)**^a^	***P*** ^c^	***β*** **(SE)**^a^	***P*** ^d^
**Main effects**						
With vs. without depressive symptoms at 3^rd^ trimester^b^	0.020 (0.068)	0.771	0.192 (0.116)	0.098	0.140 (0.179)	0.435
Postpartum days	−0.001 (0.001)	0.053	0.002 (0.001)	0.006	−0.002 (0.002)	0.332
**Interaction term** Postpartum days # With vs. without depressive symptoms at 3^rd^ trimester	−0.001 (0.001)	0.194	−0.001 (0.002)	0.445	−0.003 (0.004)	0.455

a *Longitudinal mixed-effect model with beta coefficients (β) and standard error (SE), p-value refers to maximum likelihood estimator*.

b *Depressive symptoms were assessed by the Edinburgh Postnatal Depression Scale (EPDS), yes (EPDS ≥11) and no (EPDS <11)*.

c *Models were adjusted for maternal age, education, pre-pregnancy BMI, B-vitamin (including folate) supplement intake during pregnancy, and serum folate concentration at the 3^rd^ trimester of pregnancy*.

d *Model was adjusted for pre-pregnancy BMI, B-vitamin (including folate) supplement intake during pregnancy, and serum B-12 concentration at the 3^rd^ trimester of pregnancy. # refers to the interaction term*.

**Table 3:** Models of longitudinal prediction of maternal serum homocysteine and vitamin B-12 and milk B-12 trajectories based on the presence of anxiety symptoms at the 3^rd^ trimester of pregnancy, Rio de Janeiro, Brazil.

**Unadjusted model**	**Log serum homocysteine (mg/ L) (n = 90)**		**Log serum B-12 (pmol/L) (n = 90)**		**Log milk B-12 (pmol/L) (n = 94)**	
	***β*** **(SE)**^a^	***P***	***β*** **(SE)** ^a^	***P***	***β*** **(SE)** ^a^	***P***
**Main effects**						
With vs. without anxiety symptoms at 3^rd^ trimester^b^	0.141 (0.060)	0.021	−0.182 (0.001)	0.082	−0.251 (0.177)	0.160
Postpartum days	−0.001 (0.001)	0.313	0.000 (0.001)	0.029	−0.004 (0.002)	0.081
**Interaction term** Postpartum days # with vs. without anxiety symptoms at 3^rd^ trimester	−0.002 (0.001)	0.035	0.000 (0.000)	0.895	0.001 (0.003)	0.876
**Adjusted model**	***β*** **(SE)**^a^	***P*** ^c^	***β*** **(SE)** ^a^	***P*** ^c^	***β*** **(SE)** ^a^	***P*** ^d^
**Main effects**						
With vs. without anxiety symptoms at 3^rd^ trimester^b^	0.167 (0.064)	0.010	−0.179 (0.109)	0.104	−0.168 (0.181)	0.354
Postpartum days	−0.001 (0.001)	0.348	0.002 (0.001)	0.019	−0.004 (0.002)	0.092
**Interaction term** Postpartum days # with vs. without anxiety symptoms at 3^rd^ trimester	−0.002 (0.001)	0.024	−0.000 (0.001)	0.797	0.002 (0.004)	0.585

a *Longitudinal mixed-effect model with beta coefficients (β) and standard error (SE), p-value refers to maximum likelihood estimator*.

b *Anxiety state was assessed by the State-Trait Anxiety Inventory (STAI), yes (STAI ≥40) and no (STAI <40)*.

c *Models were adjusted for maternal age, education, pre-pregnancy BMI, B-vitamin (including folate) supplement intake during pregnancy, and serum folate concentration at the 3rd trimester of pregnancy*.

d *Model was adjusted for pre-pregnancy BMI, B-vitamin (including folate) supplement intake during pregnancy, and serum B-12 concentration at the 3rd trimester of pregnancy. # refers to the interaction term*.

## Discussion

Women with anxiety symptoms in the third trimester of pregnancy presented higher median serum homocysteine concentrations at 2–8 postpartum days and lower serum and milk B-12 at 28–45 days compared with women without anxiety symptoms. Women with anxiety symptoms exhibited a decrease in homocysteine concentrations in the first 3 months postpartum compared to women without anxiety symptoms. However, depressive symptoms did not change the postpartum trajectories of the maternal serum B12, homocysteine, and milk B-12 concentrations. Maternal serum B-12 concentration was positively correlated to milk B-12 concentration.

Pregnancy and lactation are characterized by increased nutrient requirements for women; thus, these are considered stages at which women are at higher risk of B-12 insufficiency (4). In addition, serum B-12 concentrations by themselves may not be sufficiently accurate to confirm or refute this assumption; hence other biomarkers have been suggested (4, 45). For example, homocysteine levels have been used as a sensitive marker of folate and B-12 deficiency (46). In countries with mandatory flour fortification with folic acid (such as Brazil), homocysteine is likely to be an accepted biomarker for B-12 deficiency (47). Our study observed lower median concentrations of serum B-12 and homocysteine at the end of pregnancy compared to the postpartum period, which is in line with the literature. A decrease in the B-12 (~25–30%) is expected through pregnancy, which has been assumed to be a normal physiological response (48–50). In addition, previous studies also showed that the homocysteine concentrations could increase during pregnancy; however, pregnant women have lower serum concentrations compared to non-pregnant women (48).

The studies conducted prior to ours' had yielded conflicting conclusions about the relationship between depression and B-12 or homocysteine concentrations (15). Some studies, with adults and elderly individuals, have indicated hyperhomocysteinemia in patients with depression (51, 52), while others did not find any association (53). Data from NHANES 2005–2006 showed that depressive symptoms among participants aged 20–85 years were not associated with homocysteine concentration; nevertheless, they found a positive association when they restricted the analysis to participants older than 50 years. Therefore, age could be a possible path to modifying this relationship (54). In this context, it is also important to highlight that our study sample was comprised of young (18–40 years old) women, without any case of hyperhomocysteinemia, perhaps because most of the women in our sample have taken folic acid supplements during pregnancy.

Even without any case of hyperhomocysteinemia, we observed higher median homocysteine concentrations in the first 2–8 days and lower B-12 concentrations at approximately the 1st month postpartum, respectively, in women with anxiety compared to those without anxiety. The association between B-12 or homocysteine concentration and anxiety is less investigated than this relationship with depression, and few studies were performed during pregnancy or postpartum. Regarding this result, an experimental study observed that the increased homocysteine concentration in rats might be the result of stress-induced depression rather than a cause of depression (55). However, in our study, women with anxiety presented a daily decrease in postpartum homocysteine concentration compared to women without. It is also possible that the strength of the association of maternal mental health at the end of pregnancy over B-12 and homocysteine may be stronger in the 1st days and up to the 1st month postpartum, thereafter mental health could be influenced more strongly by other environmental factors, which could explain the decrease in postpartum homocysteine concentration of women with anxiety symptoms.

There is a paucity of studies evaluating maternal mental health and milk composition. To our knowledge, only one study has previously evaluated the association with vitamin concentrations in human milk (25). That cross-sectional study focused only on vitamin B-6 and postpartum depressive symptoms, used a different instrument, and did not find any association (56). Even though maternal mental health was not associated with changes in the trajectories of postpartum serum and milk B-12 concentrations, we observed lower concentrations of serum and milk B-12 at 28–50 days postpartum in women with anxiety symptoms than women without these symptoms at the end of pregnancy. The manifestation of anxiety at the end of pregnancy may promote unhealthy lifestyles (18–20), and, therefore, it could result in poor B-12 maternal status and, thus, it may contribute to lower B-12 milk concentration.

Our finding regarding the correlation of maternal serum B-12 with milk B-12 concentrations aligns with previous studies that had already reported this relationship; i.e., B-12 status during both pregnancy and lactation is associated with the milk B-12 concentration (57). Similar to our results, Bae et al. (48) reported, based on data from a feeding trial study with 28 lactating women in the US, that milk B-12 concentration was moderately positively correlated with maternal serum B-12 concentration and also tended to be inversely correlated with maternal serum homocysteine at 5 weeks postpartum.

The strengths of this study include its prospective design and the use of blood status biomarker concentrations, which are more accurate and free of the self-reported bias related to the conventional methods of dietary assessments that could under-or overestimate maternal nutritional status. As homocysteine concentration could be impacted by other vitamin deficiencies and pathological conditions, we adjusted our models for serum folate concentration, which could impact homocysteine and B-12 concentrations and maternal mental health, and only included healthy women (without any chronic or infectious diseases) in the study. Besides, none of the participants was taking antidepressants or any medication for anxiety or depression upon enrollment.

We acknowledge that our study has some limitations. Although we adjusted our models for important confounders, we cannot rule out residual confounders by other factors, such as genetic makeup, that may be linked to both maternal mental health distress and B-12 and homocysteine concentrations. Second, although limited empirical evidence has shown that milk vitamin B-12 seems not to vary during the feeding episode, this requires further research as existing data is still conflicting. Thus, we acknowledge that B-12 milk concentration in our study could have been affected as the breast was not fully emptied, and not every child was exclusively breastfed. Finally, our study had a limited sample size and was conducted in only one public health center, limiting the generalization of our findings.

Considering that there is a high prevalence of mental health disorders during pregnancy, particularly in low- and middle-income countries (21), our study contributes evidence on the association of this condition with postpartum blood and milk vitamin B12 concentration, which are important determinants of a child's nutritional status. Vitamin B12 deficiency can start in early infancy due to low fetal storage at birth if the mother is deficient during pregnancy. Deficits can continue or be exacerbated by poor maternal vitamin B12 status during lactation with consequent low amounts of the vitamin in the human milk, increasing the risk of infant short- and long-term outcomes, such as growth and developmental delays (3, 4).

In conclusion, we observed differences in concentrations of serum B-12, homocysteine, and milk B-12 between women with and without anxiety symptoms. Our results support previous studies that showed a positive correlation between maternal serum B-12 and milk B-12 concentration. Additionally, anxiety in the third trimester of pregnancy was associated with changes in postpartum trajectories of serum homocysteine concentration. Thus, our findings suggest that monitoring maternal mental health and the biomarkers of vitamin B12 status could be an essential strategy for better maternal and child nutritional status. However, large randomized clinical trials in populations with a high prevalence of B-12 deficiency are needed to explain better the mechanisms by which maternal mental health disorders could be related to maternal serum and human milk vitamin composition.

## Data Availability Statement

The original contributions presented in the study are included in the article/Supplementary Materials, further inquiries can be directed to the corresponding author/s.

## Ethics Statement

The studies involving human participants were reviewed and approved by The Research Ethics Committee of the Municipal Health Secretary and Civil Defense of the State of Rio de Janeiro (Protocol number: 49218115.0.0000.5275) and the Maternity School of Rio de Janeiro Federal University (Protocol number: 49218115.0.0000.5275) approved the present study. The patients/participants provided their written informed consent to participate in this study.

## Author Contributions

MB, AFe, NF-C, and GK designed and conducted the research. MB analyzed data and performed statistical analysis. MB, PR, AFe, NF-C, AFi, and GK wrote the manuscript's first draft. DH performed analysis of vitamin B-12 in human milk samples. DH, SS-F, LA, and RP-E contributed to interpretation of findings and provided critical evaluation and input into the manuscript. All authors edited the manuscript and read and approved the final manuscript.

## Funding

The National Council for Scientific and Technological Development (CNPq in the Portuguese acronym; grant number: 409676/2016), the Carlos Chagas Filho Foundation for Research Support of Rio de Janeiro State (FAPERJ, grant number: E-26/210.190/2014), and intramural USDA-Agricultural Research Service project 5306-51000-004-00D funded the study. GK received a research productivity scholarship from CNPq. This study was also funded in part by the *Coordenação de Aperfeiçoamento de Pessoal de N*í*vel Superior - Brasil* (CAPES in the Portuguese acronym) - Finance Code 001.

## Conflict of Interest

The authors declare that the research was conducted in the absence of any commercial or financial relationships that could be construed as a potential conflict of interest.

## Publisher's Note

All claims expressed in this article are solely those of the authors and do not necessarily represent those of their affiliated organizations, or those of the publisher, the editors and the reviewers. Any product that may be evaluated in this article, or claim that may be made by its manufacturer, is not guaranteed or endorsed by the publisher.
