# Supplementary material for: Maternal Mental Health in Late Pregnancy and Longitudinal Changes in Postpartum Serum Vitamin B-12, Homocysteine, and Milk B-12 Concentration Among Brazilian Women
Source: Front Nutr. 2022 Jul 11;9:923569. doi: 10.3389/fnut.2022.923569 (PMC9309881; doi:10.3389/fnut.2022.923569)
Supplement: Supplementary file 1 [file Data_Sheet_1.docx]

Supplementary Material

**Supplementary Figure 1.** Fluxogram of women with postpartum biological samples followed in the cohort in Rio de Janeiro, Brazil.

| **Supplementary Table 1**. Comparison of baseline characteristics of women with and without biological samples, Rio de Janeiro, Brazil. | | | | | | | |
| --- | --- | --- | --- | --- | --- | --- | --- |
|  | **Women**  **with biological samples**  **(n=101)** | | **Women**  **without biological samples**  **(n=34)** | | |  | |
|  | n | % | | n | % | | p^1^ |
| Maternal age (years) |  |  | |  |  | | 0.97 |
| <30 | 68 | 67.3 | | 23 | 67.5 | |  |
| ≥ 30 | 33 | 32.7 | | 11 | 32.4 | |  |
| Education (years) |  |  | |  |  | | 0.76 |
| ≤ 12 | 88 | 88.0 | | 31 | 91.2 | |  |
| > 12 | 12 | 12.0 | | 3 | 8.8 | |  |
| Marital status |  |  | |  |  | | 0.62 |
| With a partner | 84 | 83.2 | | 27 | 79.4 | |  |
| Without a partner | 17 | 16.8 | | 7 | 20.6 | |  |
| Depressive symptoms^2^ |  |  | |  |  | | 0.79 |
| Yes (≥11) | 36 | 35.6 | | 13 | 38.2 | |  |
| No (<11) | 65 | 64.4 | | 21 | 61.8 | |  |
| Anxiety state^3^ |  |  | |  |  | | 0.87 |
| Yes (≥40) | 40 | 39.6 | | 14 | 41.2 | |  |
| No (<40) | 61 | 60.4 | | 20 | 58.8 | |  |
| ^1^Chi-square or Fisher’s exact test  ^2^ Depressive symptoms were assessed by the Edinburgh Postnatal Depression Scale (EPDS) in 3^rd^ trimester of pregnancy.  ^3^ Anxiety state was assessed by the State-Trait Anxiety Inventory (STAI) in the 3^rd^ trimester of pregnancy. | | | | | | | |
